# Gastric Candidiasis in Five Horses: A Case Series

**DOI:** 10.3390/microorganisms13081746

**Published:** 2025-07-25

**Authors:** Patricia Neira-Egea, Clara Alamar Malvoisin, María de la Cuesta-Torrado, Claudia Bautista-Erler, Valentina Vitale, Sandra Jolly, Carla Cesarini

**Affiliations:** 1 Department of Animal Medicine and Surgery, Institute of Biomedical Sciences, Cardenal Herrera-CEU University, CEU Universities, 46115 Alfara del Patriarca, Spain; patricia.neiraegea@uchceu.es (P.N.-E.); maria.de2@uchceu.es (M.d.l.C.-T.); claudia.bautistaerle@uchceu.es (C.B.-E.); valentina.vitale@uchceu.es (V.V.); 2 Equine Clinical Department & Fundamental and Applied Research for Animals & Health (FARAH), Faculty of Veterinary Medicine, University of Liège, Bât. B41, 4000 Liège, Belgium; cmalvoisin@uliege.be; 3 Laboratoire D’anatomopathologie (Histopathologie et Cytologie), Business Village Ecolys, Avenue d’Ecolys 2, Boite 42, Suite 210, 5020 Suarlée, Belgium; anapath.be@antechdx.com

**Keywords:** equine, *Candida*, stomach, yeast, gastrointestinal, colic, ulcer, omeprazole, inflammatory bowel disease, microbiota

## Abstract

*Candida* spp. are ubiquitous yeasts that are part of most mammals’ microbiota and can become opportunistic pathogens under predisposing conditions. Interestingly, recent studies in human medicine report an increased abundance of *Candida* spp. in association with inflammatory bowel disease (IBD). Gastrointestinal candidiasis has been primarily reported in neonatal foals, but not in adult horses. The aim of this study is to describe the morphological, histopathological, and microbiological features of gastric lesions associated with *Candida* infiltration in five horses referred to two tertiary hospitals for different reasons. Clinical features, findings from gastroscopy, gastric, and duodenal biopsies, as well as fungal and bacterial cultures obtained from gastric lesions will be reported. Macroscopically, gastric lesions showed a characteristic yellow/white pseudo-membranous appearance, similar to lesions reported in foals. The presence of *Candida* spp. was confirmed by positive culture and/or histopathological evidence of fungal infiltration on the gastric epithelium. Three out of five horses showed histopathological changes in duodenal biopsies, potentially suggesting IBD. These results demonstrate that gastric candidiasis can occur in adult horses. Further research is needed to elucidate the pathogenesis, predisposing factors, and clinical relevance of *Candida* spp. infections in the equine stomach, as well as their potential impact on gastrointestinal health and overall performance.

## 1. Introduction

*Candida* spp. are ubiquitous yeasts that naturally colonize the skin and mucosal surfaces of the gastrointestinal, upper respiratory, and lower urogenital tracts in most mammals [1,2]. Under normal conditions, an individual’s microbiota prevents excessive proliferation.

However, certain risk factors may predispose the host to *Candida* spp. overgrowth and colonization. These include immunosuppressive treatments, prolonged antibiotic therapy, severe illness, primary or acquired immunodeficiency, nutritional deficiencies, and the disruption of the cutaneous and gastrointestinal barriers [1,3,4,5]. Interestingly, recent studies in human medicine report an increased abundance of *Candida* spp. in association with inflammatory bowel disease (IBD), although further research is necessary to clarify the potential role of fungi in this condition [6].

The gastrointestinal tract is one of the anatomical locations where candidiasis most commonly develops in humans, together with the skin and the genitourinary tract [7]. In other animal species, gastrointestinal cases have also been described [8,9,10]. In adult horses, cases of candidiasis affecting the eyes, respiratory tract, urogenital system, and joints have been documented, primarily in individuals that are immunocompromised or receiving antimicrobial treatment [11,12,13,14]. In the equine species, gastrointestinal candidiasis has only been reported in foals, affecting different locations such as the mouth, esophagus, and stomach [15]. Other manifestations of candidiasis described in foals include diarrhea, meningitis, omphalophlebitis, and, less frequently, systemic infections [1,16].

Five horses were referred for different reasons to two tertiary referral hospitals, the CEU Veterinary Clinical Hospital in Spain (Case 1) and the Equine Hospital of the University of Liège in Belgium (Cases 2–5) between July 2023 and July 2024. As part of the individual work-up of each case, a gastroscopy was performed, and gastric candidiasis was confirmed by culture and/or histopathology. This case series aims to describe the morphological, histopathological, and microbiological features of this condition which, to our knowledge, has not previously been described in adult horses. The clinical findings, diagnosis, and management of the five cases, as well as potential predisposing factors for the fungal infection, will be discussed.

## 2. Case Description

### 2.1. Case 1

A 10-year-old KWPN gelding used for dressage was referred to a veterinary hospital for an investigation of chronic weight loss. The clinical examination was within normal limits, and its body condition score (BCS) was 4/9 following the Henneke scale [17].

Initial blood work revealed mild neutropenia and lymphopenia, as well as a mild increase in gamma-glutamyl transferase (GGT) serum enzyme activity. An abdominal ultrasound revealed distended and moderately thickened (4–6 mm) small intestine loops in both inguinal regions and a mild accumulation of peritoneal fluid. Fecal culture and fecal egg count, abdominal radiology, thymidine kinase, and a glucose absorption test (85%) were within normal limits. 

**Figure 1:**
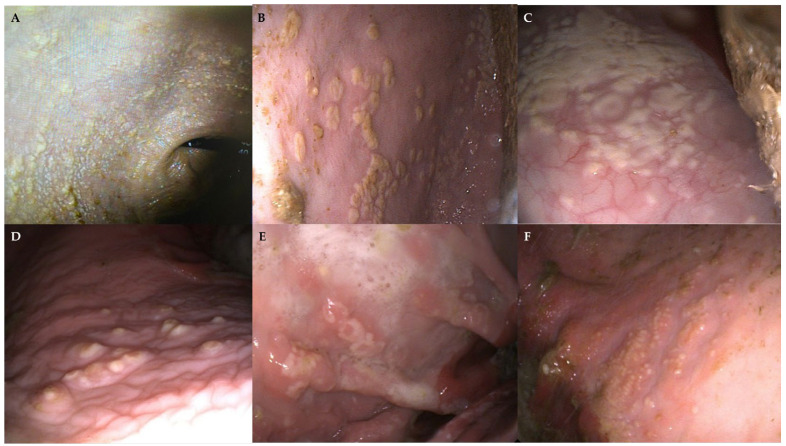
Gastroscopic images showing white, cotton-like material on the squamous mucosa of horses with gastric candidiasis, forming pseudo-membranous multifocal to coalescing plaques (**A**–**C**). In some cases, fungal lesions appeared punctiform (**D**), ring-shaped (**E**) or with a miliary appearance (**F**), close to the greater curvature. An underlying hyperemic area, extending beyond the plaques, is visible in some lesions (**E**,**F**).

As part of the weight loss work-up, a gastroscopy was performed, and atypical, white, easily detachable cotton-like patches on the squamous gastric mucosa were observed (Figure 1). Grade 2 ulcers were observed on the squamous gastric mucosa, and diffuse hyperemic areas were visible in the glandular portion and duodenum (Table 1) [18].

Gastric and duodenal biopsies were obtained as part of the weight loss investigation. Histopathologic evaluation revealed moderate gastritis and mild lymphoplasmacytic enteritis (Table 2). The culture of the gastric lesions was positive for *Candida* spp., along with *Klebsiella pneumoniae*, *Pseudomonas mendocina*, and *Acinetobacter baumannii*.

The horse was diagnosed with equine gastric ulcer syndrome (EGUS) and potential lymphoplasmacytic enteritis. It was discharged under treatment with omeprazole, sucralfate, and probiotics (Menarini Consumer Health, Barcelona, Spain; TRM, Newbridge, Ireland) as well as the recommendation of a controlling gastroscopy one month later. Additional treatment with fluconazole was recommended but was never administered by the owner. A tapering protocol with corticosteroids was recommended after gastric candidiasis resolved.

After discharge, the horse left the country and was lost to follow-up. When contacted one year later, the owner reported that the follow-up gastroscopy had been performed five months after discharge at another clinic. Complete resolution of the gastric ulcers was observed, with no remaining evidence of the initial fungal, cotton-like lesions.

### 2.2. Case 2

A 3-year-old miniature stallion was admitted to the veterinary hospital with signs of lethargy that had been developing for one week. There was also a history of progressive weight loss in the last 2 years. On arrival, the pony was dull and hypothermic (36.9 °C) with an estimated BCS of 3/9.

Blood analysis showed mild anaemia, hypoalbuminemia and hyperlactatemia.

After initial stabilisation (Lactated Ringer’s, intravenous glucose, and plasma, B. Braun, Melsungen, Germany), several ancillary tests were performed during hospitalization to investigate the cause of weight loss, including dental examination, fecal egg count, abdominal/thoracic radiology and ultrasound, peritoneal fluid cytology, oral glucose tolerance test (OGGT), and a gastroscopy (day 10), including duodenal biopsies. Given its poor appetite, the horse had been started on proton pump inhibitors (omeprazole 2 mg/kg PO q24 h, Norbrook Laboratories, Monaghan, Ireland) one day before gastroscopy.

The gastroscopy revealed mainly atypical large multifocal coalescent plaques of a friable white pseudo-membranous material located on the squamous gastric mucosa (Figure 1B,C). A complete description of gastroscopic findings can be found in Table 1. The aspect of the plaques evoked lesions of oral candidiasis, so a fungal aetiology was suspected. Biopsies were performed and sent for fungal culture and histopathology, yielding positive results for *Candida* spp. Duodenal biopsies were obtained as well, and the results confirmed a mild lymphoplasmacytic infiltrate. Detailed histopathological results are described in Table 2.

A presumptive diagnosis of lymphoplasmacitic enteritis associated with partial malabsorption (45%) was concluded. Given the strong suspicion of IBD, a decreasing protocol of corticosteroids (initially dexamethasone (Dechra Veterinary Products, Herentals, Belgium) starting at 0.1 mg/kg IM q24 h for 15 days and prednisolone (Le Vet.B.V., Oudewater, The Netherlands) at 2 mg/kg PO q24 h over several weeks) was implemented, and omeprazole therapy was continued.

One week later, before leaving the hospital (day 18), a second gastroscopy was performed, revealing the complete resolution of the fungal plaques.

### 2.3. Case 3

An 11-year-old crossbred mare was referred to the veterinary hospital due to an acute onset of hyperthermia (41 °C) 2 weeks earlier, followed by inappetence. Weight loss had been noticed over the last few months, taking place after the mare moved to a new barn. Suspecting piroplasmosis, the referring veterinarian had administered corticosteroids and imidocarb 5 days prior to admission.

On clinical examination, the mare showed a BCS of 3/9 with generalised muscular atrophy. Mild icterus, a heart rate of 48 bpm, and a rectal temperature of 36.9 °C were also noticed.

The blood analysis revealed moderate neutrophilic leucocytosis, mild hypoalbuminemia, increased serum amyloid A (SAA), and hypovitaminosis E. Direct polymerase chain reaction (PCR) blood testing for *Theileria equi* and *Babesia caballi* yielded negative results. A left dorsal displacement of the large colon was identified on the rectal examination. The weight loss work-up revealed no abnormalities on dental examination, fecal egg count, thoracic/abdominal imaging (US and X-Ray), or abdominal fluid cytology.

**Figure 2:**
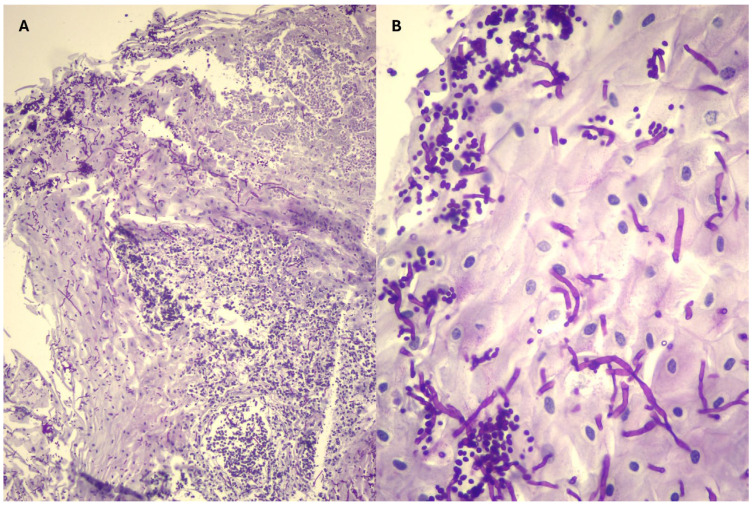
Histopathological images of the endoscopic biopsies taken from the white plaques on the squamous mucosa of horses with gastric candidiasis. Periodic acid-Schiff stain. (**A**) Squamous epithelium with fungal spores and hyphae, pyocytes, and bacteria (10×). (**B**) Numerous spores and hyphae colonizing the squamous epithelium of the equine stomach (40×).

On gastroscopy, the stomach showed a few atypical pearl-shaped patches on the non-glandular mucosa (Table 1). Biopsies of the patches were performed, and, despite no growth on the fungal culture, the histopathology confirmed fungal infiltration (Figure 2). Duodenal biopsies were obtained as well. Detailed histopathological results can be found in Table 2.

No more fever was noted, and the mare gained weight during hospitalization, so a presumptive diagnosis of transient viral infection and inadequate food intake were concluded. The mare left the clinic without any treatment except for dietary recommendations.

No further gastroscopies were performed in this case. Several months after hospitalisation, the mare had recovered an adequate body condition (6/9) and was reported to be clinically normal.

### 2.4. Case 4

A 9-year-old Friesian crossbred mare was referred to the veterinary hospital with signs of colic.

The mare had a BCS of 7/9 and presented tachycardia (58 bpm) and reduced borborygmi on admission.

Clinical chemistry evaluation revealed hyperlactatemia and a moderate neutrophilic leucocytosis. The large colon felt tympanic and was displaced caudally to the right on rectal examination. A right colon displacement was diagnosed and treated successfully with intravenous (Lactated Ringer’s, B. Braun, Melsungen, Germany) and enteral fluid therapy and 24 h of food withholding. On day 2, the mare presented a transient phase of soft feces, fever, and neutropenic leukopenia without further clinical signs. A Fecal PCR was positive for *Salmonella* spp. Activated charcoal (Vinoferm, Beverlo, Belgium), smectite (Platinum Performance, Buellton, CA, USA), flunixin meglumine (0.25 mg/kg q6 h, Syva, León, Spain), and enoxaparin (Sanofi-Aventis, Argenteuil, France) were added to the treatment.

The mare presented poor appetite during refeeding. Suspecting EGUS, omeprazole (Norbrook Laboratories, Monaghan, Ireland) and sucralfate (EXOD, Saint-Cloud, France) were started on day 4, and a gastroscopy was performed on day 6. Atypical white, ring-shaped plaques were visible in the squamous gastric mucosa, some of them surrounded by an erythematous halo (Table 1). Biopsies of the plaques were performed and sent for fungal culture and histopathology, confirming the presence of *Candida* spp. in the lesions. Significant pyloric ulcers were confirmed and biopsied as well. Detailed results of the histopathological exam can be found in Table 2.

The horse was discharged on treatment with omeprazole (4 mg/kg PO q24 h) and sucralfate (12 mg/kg PO 12 h) for 3 weeks, and a follow up gastroscopy was planned.

The appetite of the mare had normalized during treatment, and the second gastroscopy showed a significant improvement of the pyloric and squamous lesions. Although the white plaques were mostly gone, some of the initial erythematous lesions remained on the squamous mucosa. The nature and clinical significancy of the remaining lesions not being clear, the owner’s consent was obtained to take additional gastric and duodenal biopsies. Detailed histopathological results can be found in Table 2. The treatment with omeprazole and sucralfate was continued for 3 additional weeks to ensure the complete resolution of the pyloric ulcers.

### 2.5. Case 5

A 2-year-old purebred Lusitanian mare was found stuck in a flooded field contaminated by fuel after local torrential rains. The first veterinarian who cared for it washed the skin and the stomach, administered activated charcoal and mineral oil by nasogastric tubing, and referred the mare to the veterinary hospital for further care and follow up.

On arrival, the mare showed a BCS of 5/9 and was agitated, tachycardic (56 bpm), and presented several superficial wounds all over the body.

Blood analysis showed neutrophilic leucocytosis and increased creatine phospho-kinase (CPK) activity. Thoracic radiology was normal, but lung ultrasonography showed abnormal B-lines, so early-stage pneumonia could not be excluded. Abdominal ultrasound showed no abnormalities.

The mare was placed on intravenous fluid therapy with Lactated Ringer’s (B. Braun, Melsungen, Germany), large spectrum antimicrobials (natrium penicillin 22,000 IU/kg IV q6 h (Kela, Hoogstraten, Belgium) and gentamicin 6.6 mg/kg IV q24 h (Emdoka bvba, Hoogstraten, Belgium)), NSAIDs (flunixin meglumine 1.1 mg/kg IV q12 h, Syva, León, Spain), enoxaparin (Sanofi-Aventis, Argenteuil, France), activated charcoal (Vinoferm, Beverlo, Belgium), smectite (Platinum Performance, Buellton, CA, USA), and appropriate wound care. The stomach and the skin showed fuel residues and were washed further, and the mare was kept hospitalized under observation for several days. Evolution was good, with no abnormal clinical signs except for transient soft feces.

On day 8, a gastroscopy was carried out to evaluate for potential gastrointestinal injury secondary to hydrocarbon ingestion. Squamous and glandular gastric ulcers were found (see detailed description in Table 1). Furthermore, atypical multifocal miliary white vesicles surrounded by an erythematous halo were observed on the squamous mucosa, which were not related to the more “classical” gastric ulcers (Figure 1F). Biopsies were performed on those atypical lesions and on the duodenum (see detailed description in Table 2). Fungal culture confirmed the presence of *Candida glabrata*.

A treatment with omeprazole (4 mg/kg PO q24 h) and sucralfate (12 mg/kg PO q12 h) was started, and the mare was discharged (Day 10).

A follow-up gastroscopy was performed 4 weeks later by the referral veterinarian. The gastric ulcers had almost resolved, and the whitish lesions were no longer visible.

## 3. Discussion

This study describes for the first time the findings associated with gastric candidiasis in adult horses. Previously, equine gastrointestinal candidiasis had only been reported in foals. In the five cases described in this report, all adult horses exhibited similar lesions (cotton-like plaques, easily detachable) on the non-glandular gastric mucosa, very similar to lesions observed in the oral or gastrointestinal mucosa of neonatal foals [1,16,19,20]. Despite the extensive experience of the authors in equine gastroscopic examination, such lesions had never been previously observed in adult horses. For that reason, cases were analyzed regarding literature and to look for common features or predisposing factors.

*Candida* spp. is a microorganism that naturally exists within the saprophytic mycobiome but can act as an opportunistic pathogen in favorable conditions. In human medicine, studies describe the presence of candidiasis in patients with imbalances in saprophytic flora, antibiotic therapy, chemotherapy, radiotherapy, stress, or immunosuppression therapy [5]. An increased incidence of candidiasis has been reported in adult horses treated with antibiotics or corticosteroids or that were in states of immunosuppression, as well as in foals with a failure of passive immunity transfer [19,21]. Three cases were presented for weight loss, although in at least one of them, the cause was found to be malnutrition. Only one horse out of the five (Case 5) had received antimicrobials (penicillin and gentamicin) before gastroscopic examination. Additionally, another horse (Case 3) had received a single dose of corticosteroids. In which degree these treatments may have helped *Candida* overgrowth in these two cases remains unknown.

Some articles in human medical literature have investigated the potential relationship between the use of gastric acid suppressors and gastrointestinal fungal overgrowth. Gastric acid is considered a main barrier for the inhibition of fungal and bacterial overgrowth of the stomach and small bowel, and its absence has been associated with gastroenteritis caused by *Clostridium difficile*, *Salmonella*, *and Campylobacter* as well as with rapid increases in *Candida* concentrations in gastrointestinal content [22]. It has been shown that the use of gastric acid suppressor therapy may promote increased *Candida* colonization in the esophagus and stomach [22,23,24]. Interestingly, the concurrent use of proton pump inhibitors and steroids in the same patient substantially increased the risk of esophageal candidiasis compared to the effect of each medication alone, suggesting a synergistic effect [25]. In animals, few studies have been published on the relationship of gastric acid inhibition and gastrointestinal colonization by *Candida* spp. A study in rats showed that previous treatment with antisecretory agents or non-steroidal anti-inflammatory drugs allowed persistent colonization with *Candida* after experimental inoculation [26]. From the five horses included in this study, only two (Case 2 and Case 4) had received gastric protectants or gastric acid suppression prior to gastroscopy. Given the short course of the therapy before gastroscopy in both cases (1 and 4 days, respectively), it is unlikely that they played a major role in the development of the gastric lesions observed. Furthermore, it is interesting to notice that gastric fungal lesions resolved in three of the five horses while receiving antiulcer treatment (omeprazole and/or sucralfate) for the prevention or treatment of concomitant EGUS.

Traditionally, the stomach was considered a sterile environment; however, it is now known that both in humans and animals there is a gastric microbiota [27]. It is important to note that the composition of the gastric microbiota can vary based on external factors such as diet, transportation, thermal stress, high performance, age, geographic location, medical treatments, and the presence of diseases. These factors can significantly influence the microbial balance of the gastrointestinal tract [28]. Although the gastric microbiota in horses has not been widely characterized, the presence of genera such as *Lactobacillus* spp., *Streptococcus* spp., *Actinobacillus* spp., *Moraxella* spp., *Prevotella* spp., and *Porphyromonas* spp. has been demonstrated through samples taken from healthy patients. In the gastrointestinal tract, *Candida* spp. can overgrow alongside other pathogenic microorganisms such as *Clostridium difficile*, *Pseudomonas* spp., *Helicobacter* spp., *Enterobacter* spp., and *Escherichia coli* [29]. In Case 1, a bacteriologic study of gastric white plaques was performed, and the growths of *Klebsiella pneumoniae*, *Pseudomonas mendocina*, and *Acinetobacter baumannii* were observed. Despite no bacterial cultures of gastric lesions being performed in Cases 2–5, histopathology revealed often a suppurative gastritis, including neutrophilic infiltration and the presence of bacteria. The interpretation of this data in relation to the presence of fungal lesions is difficult. Despite the fact that some of those bacteria could be merely contaminants, in humans, it has been observed that the excessive growth of *Actinobacillus* spp, *Pseudomona* spp., and *Helicobacter* spp. under certain conditions may be associated with gastric disorders, and something similar could happen in horses. This finding opens the door to future research in veterinary medicine that could explore the role of these pathogens in the gastric health of animals [27,28]. Stomach and gastric feeding tubes have been frequently found colonized by oral *Candida* spp. in human patients [30]. *Candida* spp. have been identified as part of the normal nasal microbiota in healthy horses [2]. This raises the question of whether nasogastric intubation could promote *Candida* spp. proliferation in horses. Four out of the five patients reported in this study underwent nasogastric tubing for enteral fluid therapy for less than 24 h prior to the identification of gastric lesions associated with *Candida* spp. Mechanical irritation from the tube could have acted as an initial insult, facilitating fungal proliferation. Although this hypothesis cannot be entirely ruled out, it seems unlikely to account for the extent and distribution of the fungal lesions observed in these cases.

The diagnosis of *Candida* spp. infections is usually confirmed through histopathology or fungal culture of affected tissues and exudates. Among the species responsible for candidiasis, *Candida albicans* remains the most prevalent etiological agent, followed by *C. glabrata*, *C. tropicalis*, *C. parapsilosis*, *and C. krusei* [1,6,31]. Depending on the species, different morphological forms—yeasts, pseudo-hyphae, or hyphae—can be observed [8,32]. From the five cases reported here, *C. glabrata* was isolated in one of them (Case 5). Unfortunately, the laboratories did not go further in the species identification of the other isolated *Candida*. Case 3 yielded negative fungal culture results, but histopathological findings supported fungal infection of the lesions (Table 2).

Human research has identified an association between *Candida albicans* colonization and various inflammatory disorders of the gastrointestinal tract, suggesting that such colonization might hinder the resolution of inflammatory responses [7]. Findings from animal model studies indicate that *Candida* colonization impairs the healing of inflammatory lesions, while inflammation itself can enhance fungal colonization. In human medicine, fungal growth has commonly been described in both non-glandular and glandular (peptic) gastric ulcers [33]. Gastric candidiasis in foals has also been associated with gastric ulcers in the non-glandular mucosa [15,20]. Inflammation of the gastric mucosa may be linked to a decline in local bacterial populations, facilitating fungal proliferation [7]. In the present report, two horses had grade-2 squamous ulcers (Cases 1 and 5), and three horses had different kinds of glandular ulcers (Cases 1, 4, and 5), but mycotic lesions did not seem associated with those ulcerative lesions in any of the five cases. Interestingly, erythematous mucosa underlying *Candida* lesions has been described in human oral candidiasis when the characteristic white plaques are wiped away [34]. In some of the cases reported here, we observed an erythematous halo surrounding the fungal lesions (Figure 1E,F).

Inflammatory bowel diseases (IBDS) are a group of gastrointestinal tract disorders characterized by the infiltration of the mucosa and submucosa with inflammatory cells [35]. Consequently, there may be an alteration in the microbiome, which can contribute to an overgrowth of fungal elements [36]. In human medicine, indeed, IBD has been associated with the increased growth of *Candida* spp., which can further compromise the gastrointestinal barrier, heighten inflammation, and slow down tissue healing [7]. Only one of the five horses (Case 1) presented macroscopic lesions in the duodenal area; however, duodenal biopsies were taken in all of them to investigate the potential presence of IBD together with *Candida* spp. colonization. In three of them, there was a history of weight loss and histological duodenal findings of mild to moderate lymphoplasmacytic inflammatory bowel disease (Cases 1, 2, and 3). One of them presented distended and thickened small intestinal loops as well (Case 1). However, in the other two cases (Cases 4 and 5), IBD was not supported by either clinical or laboratory findings; thus, the link between *Candidiasis* and this gastrointestinal disorder currently seems weak in horses, and a potential cause-and-effect relationship remains to be elucidated.

Finally, it is interesting to notice that, in all five horses, we had evidence of improvement or even complete resolution of the fungal lesions without the need to administer any antifungal treatment. An absence of fungi in the squamous mucosa was also confirmed histologically in the control gastroscopy of one case (Case 4). Four of the five horses received antiulcer treatment (one omeprazole only and three omeprazole + sucralfate) for the prevention or treatment of concomitant EGUS. Human literature is controversial regarding the response of gastric *Candida* infection to acid suppression therapy. As already mentioned, some studies consider gastric acid suppression as a predisposing factor for fungal development [22,23,24], while others report the healing of gastric ulcers colonized by *Candida* after 6 weeks of proton pump inhibitor therapy only without antifungal medication [37]. Some limitations of the present case series include the lack of information about *Candida* spp. subspecies and the absence of follow-up gastroscopy in some cases.

## 4. Conclusions

In conclusion, gastric candidiasis occurs in adult horses and seems to exclusively affect the squamous gastric mucosa. Macroscopical findings look fairly similar to gastrointestinal candidiasis in foals. Diagnosis can be achieved by the observation of characteristic cotton-like multifocal plaques in gastroscopic examination or through fungal identification in culture or biopsy. No direct association with EGUS lesions has been found despite the fact that concomitant EGUS lesions may be present in affected horses. A potential association with gastric bacterial dysbiosis or intestinal inflammation in horses deserves further study. Based on the five cases presented in this series, no clear predisposing factors can be suggested for gastric candidiasis in adult horses, but the condition seems benign and resolves without need of antifungal therapy.

## Figures and Tables

**Table 1:** Detailed macroscopic findings found in the squamous mucosa, glandular mucosa, and the duodenum during gastroscopies performed to the 5 horses.

	Macroscopic Findings		
	Squamous Mucosa	Glandular Mucosa	Duodenum
Case 1	White, cotton-like material Ulcers grade 2/4	Hyperemic areas in the antrum, pylorus, and duodenum	Hyperemic areas and duodenal reflux
Case 2	Friable white pseudo-membranous multifocal to coalescing plaques	Normal	Hyperemic areas
Case 3	Two dispersed white, pearl-shaped, well-demarcated pseudomembranous patches	Normal	
Case 4	White ring-shaped and multifocal pseudo-membranous friable plaques, most of them being closer to the greater curvature. An underlying hyperaemic area, extending beyond the plaques, was visible.	Linear raised fibrinosuppurative ulcer that originated from the pyloric region and extended towards the antrum	Presence of two parasites (*Anoplocephala* spp.)
Case 5	White multifocal miliary vesicles surrounded by a reddish halo. Several ulcers grade 2/4 at the level of the greater and lesser curvature close to the margo plicatum.	Two severe fibrinohemorragic and depressed ulcers adjacent to the pylorus, one of them with fibrinosuppurative centre	Normal

**Table 2:** Detailed fungal culture and histopathology results performed from gastric and duodenal biopsies in the five horses.

Cases	Gastric Biopsy	Duodenal Biopsies	Culture
1	Squamous epithelium without hyperkeratosis	Mild lymphoplasmacytic infiltrate	*Candida* spp.
2	Squamous epithelium without hyperkeratosis with formation of micro-pustules and dilated necrotic-suppurative material. PAS staining showed heavy infiltration of the surface layers by hyphae.	Lymphoplasmacytic infiltrate (mild in the lamina propria) with marked villous atrophy, erosions, with cryptid lesions. Bacterial proliferation on the surface, with no evidence of fungal elements.	*Candida* spp.
3	Normal squamous epithelium and necrotic-purulent material with mycotic elements (hyphae) and bacteria. Rare hyphae identified by PAS staining.	Mild to moderate lymphoplasmacytic infiltrate with bacterial proliferation laying on top. No fungal, parasitic or fungal or elements in the PAS staining.	No growth
4	1st gastroscopy: Hyperkeratotic epithelium with hyphae or pseusohyphae, which appeared branched but not really segmented. There were often budding yeasts with a narrow base (suggesting *Candida*). Neutrophilic inflammation and bacterial colonisation (mostly bacilli and cocci). Pyloric lesion: Ulcerated surface with abundant fibrinopurulent material. The PAS staining did not reveal any mycosis. Giemsa staining did not reveal any significant bacterial flora. 2nd gastroscopy: Thick squamous epithelium with regular well differentiated cells. Some superficial bacteria. No inflammatory infiltrate. No fungal elements on PAS staining.	Second gastroscopy: Normal duodenal epithelium	*Candida* spp.
5	Hyperkeratosis infiltrated by a purulent inflammatory reaction and mycotic proliferation (hyphae and pseudohyphae).	Normal duodenal epithelium	*C* *andida glabrata*

## Data Availability

The original contributions presented in this study are included in the article. Further inquiries can be directed to the corresponding author.

## Abbreviations

The following abbreviations are used in this manuscript:

BCS	Body Condition Score
KWPN	Koninklijk Warmbloed Paardenstamboek Nederland
EGUS	Equine Gastric Ulcer Syndrome
PCR	Polymerase Chain Reaction
NSAIDs	Non-Steroidal Anti-inflammatory Drugs
GGT	Gamma-Glutamyl Transferase
CPK	Creatin Phosphokinase
IBD	Inflammatory Bowel Disease
